# Genetic Diversity and Mutation Frequency Databases in Ethnic Populations: Systematic Review

**DOI:** 10.2196/69454

**Published:** 2025-08-11

**Authors:** Shumaila Khan, Mahmood Alam, Iqbal Qasim, Shahnaz Khan, Wahab Khan, Orken Mamyrbayev, Ainur Akhmediyarova, Nurzhan Mukazhanov, Zhibek Alibiyeva,

**Affiliations:** 1 Department of Computer Science, University of Science and Technology Bannu, Bannu Township, Bannu, 28100, Pakistan, 92 3329959220; 2 Faculty of Business, Law and Social Sciences, Birmingham City University, Birmingham, United Kingdom; 3 Hertfordshire Business School, University of Hertfordshire, Hatfield, United Kingdom; 4 Department of Chemistry, University of Science and Technology Bannu, Bannu, Pakistan; 5 Faculty of Computing and Informatics, Multimedia University, Cyberjaya, Malaysia; 6 Institute of Information and Computational Technologies, Satbayev University, Almaty, Kazakhstan; 7 Institute of Automation and Information Technologies, Satbayev University, Almaty, Kazakhstan

**Keywords:** ethnic-specific mutation frequency databases, genetic diversity, mutation disorder, inherited disease

## Abstract

**Background:**

National and ethnic mutation frequency databases (NEMDBs) play a crucial role in documenting gene variations across populations, offering invaluable insights for gene mutation research and the advancement of precision medicine. These databases provide an essential resource for understanding genetic diversity and its implications for health and disease across different ethnic groups.

**Objective:**

The aim of this study is to systematically evaluate 42 NEMDBs to (1) quantify gaps in standardization (70% nonstandard formats, 50% outdated data), (2) propose artificial intelligence/linked open data solutions for interoperability, and (3) highlight clinical implications for precision medicine across NEMDBs.

**Methods:**

A systematic approach was used to assess the databases based on several criteria, including data collection methods, system design, and querying mechanisms. We analyzed the accessibility and user-centric features of each database, noting their ability to integrate with other systems and their role in advancing genetic disorder research. The review also addressed standardization and data quality challenges prevalent in current NEMDBs.

**Results:**

The analysis of 42 NEMDBs revealed significant issues, with 70% (29/42) lacking standardized data formats and 60% (25/42) having notable gaps in the cross-comparison of genetic variations, and 50% (21/42) of the databases contained incomplete or outdated data, limiting their clinical utility. However, databases developed on open-source platforms, such as LOVD, showed a 40% increase in usability for researchers, highlighting the benefits of using flexible, open-access systems.

**Conclusions:**

We propose cloud-based platforms and linked open data frameworks to address critical gaps in standardization (70% of databases) and outdated data (50%) alongside artificial intelligence–driven models for improved interoperability. These solutions prioritize user-centric design to effectively serve clinicians, researchers, and public stakeholders.

## Introduction

### Background

Recent advancements in genomic techniques, such as next-generation sequencing and clustered regularly interspaced short palindromic repeats technology, have revolutionized the identification of gene mutations associated with disease, enabling precise disease diagnosis and personalized treatment strategies. Completing the human genome sequence played a significant role in detecting gene mutations that cause diseases, collaborating with the emerging field of genomic medicine [1,2]. However, genetic mutations and DNA sequence alterations can disorder normal gene function and lead to various syndromes. These mutations can be categorized as affecting a single gene (Mendelian), multiple (general) genes, or a population or ethnic group (national/ethnic), with significant health implications [3].

Mutation databases are critical web-based repositories that aggregate genomic variant data for specific populations or ethnic groups, enhancing the understanding of genetic diversity and its association with the disease. Central databases, including Online Mendelian Inheritance in Man (OMIM) [4] and the Human Gene Mutation Database (HGMD) [5], primarily catalog published mutations and may not fully represent the genetic diversity of different populations [6,7]. On the other hand, locus-specific databases (LSDBs) focus on specific loci but may not gather information about a particular nation or ethnicity [8].

Other databases, like national and ethnic mutation frequency databases (NEMDBs), were developed to fill these gaps by recording the mutation spectrum observed for any gene (or multiple genes) associated with a genetic disorder for specific populations or ethnic groups worldwide. These databases are crucial for comprehending genetic variations related to diseases and facilitating targeted genetic testing and personalized medicine [9]. Regarding advancement in genomic analysis technologies, many NEMDBs face issues related to standardization, data quality, and accessibility. For example, the Human Genome Variation Society (HGVS) maintains a dedicated website; an inspection by the authors on March 12, 2024, revealed that while the page comprises 11 links, only 4 are functional, as compared to LSDBs, which contain 1646 links, and the total number of mutations was found to be 145,964. Most NEMDBs are outdated and have limited content, hindering their effectiveness in clinical and research settings [10].

Given the reliance of researchers and health care professionals on internet-enabled tools for accessing mutation data, there is a need for engineering-driven solutions to enhance further database accessibility, data standardization, and cross-platform data integration. This paper addresses the challenges by proposing an artificial intelligence–driven mutation prediction model and the linked open data (LOD) frameworks to improve data sharing, query efficacy, and interoperability within gene databases. By focusing on web-based user-centric designs, the objective is to optimize the usability of NEMDBs for health care professionals, researchers, and the general public, thereby advancing digital health solutions and improving outcomes in genetic research. By identifying the challenges and limitations associated with NEMDBs, we seek to provide actionable recommendations for enhancing their development and usability. The key contributions of the review are as follows:

- This systematic review examined 42 NEMDBs to a). analyze their design frameworks, methods of data collection, and querying capabilities; and b). identified critical gaps, including 70% (29/42) lack standardized formats, 60% (25/42) lack cross-ethnic comparisons, and 50% (21/42) have outdated data.
- To improve interoperability, engineering-driven recommendations include cloud platforms, artificial intelligence models, and LOD frameworks.
- A user-centric analysis to enhance accessibility for clinicians, researchers, and the public.

The rest of the article is organized as follows: the Related Works section presents a literature study and comprehensive review of available NEMDBs and other databases. The Methods section defines the systematic literature review approach and outlines the objectives. The Discussion section provides conclusions and future recommendations. Finally, the Conclusion section summarizes the review.

### Related Works

Recent scientific developments have brought about the emergence of bioinformatics, a multidisciplinary field that combines molecular biology, information technology, computer science, and mathematics to form a single discipline [11]. Bioinformatics encompasses various tasks such as database design, categorization, protein structure prediction, RNA folding, and mutation mapping. These systems are essential for organizing and managing biological data within structured and persistent databases critical in retrieving, updating, storing, and querying information.

A significant milestone in bioinformatics history was Margaret Dayhoff’s establishment of one of the first protein sequence databases in the 1960s; GenBank was developed in the 1980s and became the first nucleotide sequence database [12]. Similarly, mutation databases aim to make such data readily accessible to medical professionals, researchers, and clinicians studying genetic variations [13]. Recent advancements in developing integrated databases that include diverse ethnic mutation frequencies highlight the need for more inclusive data collection methods and internet-enabled platforms to bridge the genetic diversity gap [14].

PubMed, hosted by the National Center for Biotechnology Information (NCBI) since 1997, is a prominent scientific database containing several medical-related articles [15]. PubMed gives access to 38 databases concerning biomedical research and the analysis of erratic genetic diseases. Other repositories, such as MeSH (Medical Subject Headings), Institute for Scientific Information (ISI) Web of Science, and Medical Literature Analysis and Retrieval System Online (MEDLINE), provide comprehensive data about a particular gene and disease and are accessible to the public [9]. PubMed is one of the most influential bioinformatics resources, featuring web-based systems like PubMed Assistant [16], AliBaba [17], and PubMed-Ex. These enhance functionality through keyword highlighting, citation management, and semantic enrichment of biomedical entities extracted from text [18].

Similarly, the National Institutes of Health established the NCBI in 1988 as a centralized system for accessing diverse resources and databases via the NCBI website. The primary resources in the NCBI include the Database of Short Genetic Variations, the Database of Genomic Structural Variation, Entrez (an integrated database retrieval system that gives access to a diverse set of 35 databases), the Clone database (Clone DB), the BioProject Database [9,19], and the clinical central variant database (ClinVar). Table 1 summarizes the primary databases supported by NCBI, emphasizing their role in providing internet-enabled access to genomic data for researchers and health care professionals. Such internet-enabled systems streamline the extraction and analysis of gene mutation content and support collaborative research by facilitating data sharing across diverse platforms. However, challenges like data fragmentation, a lack of standardization, and accessibility limitations persist. Addressing these challenges requires leveraging artificial intelligence–based tools and LOD frameworks to improve data integration and usability. Enhancing the functionality of these systems will advance precision medicine and support clinical decision-making through electronic health applications.

**Table 1.** NCBI^a^ databases.

References	Database Name	Brief description
Bianco et al [9]	Bio Project Database	The database allows users to submit detailed research studies from intensive genome sequences projects to huge worldwide associations.
Bianco et al [9]	BioSample Database	The Biosample Database is a new resource that annotates biological samples used in various NCBI-submitted studies, including genome-wide association studies, epigenetics, genomics sequencing, and microarrays.
Landrum et al [3]	Clinical variant database (ClinVar)	ClinVar is a database that contains human genomic variants and their relevant disease. The database is publicly available.
Sayers et al [20]	PopSet Database	This database contains different sets of data that were submitted to GenBank. The data includes gene-related sequence data and their alignments with specific population, phylogenetics, mutation, and ecosystem studies.
Sayers et al [20]	Clone database (Clone DB)	The database incorporates clones and library information, including sequence data, map positions, and information distribution. It also offers filtering by organism and vector types.
Sayers et al [20]	MMDB (Molecular Modeling Database)	It details sequence alignments and profiles representing protein spheres preserved in molecule evolution.
Boguski et al [21]	Database of expressed sequence tags (dbEST) Nucleotide EST Database	This database collects sequence tags and includes details about complementary DNA (transcript) sequences. dbEST is accessible directly via the Nucleotide EST Database.
Church et al [22]	Database of Genomic Structural Variation (dbVar)	It was designed to collect details about large-scale genomic variation, including large insertions, deletions, translocations, and inversions. It also contains the relationships of different variants to their phenotype.
Louhichi et al [23]	Entrez	Entrez is a rich database that integrates information from 35 databases containing over 570 million biological data records. The database provides a graphical representation of sequences and chromosome maps, which is considered favorable in genetic research.
Mailman et al [24] and GAIN Collaborative Research Group et al [25]	Databases of Genotypes and Phenotypes (dbGaP)	The database contains information about genotype and phenotype. The information is gathered using studies such as genome-wide association studies, medical resequencing, and molecular diagnostic assays.
Sherry et al [26]	Database of Short Genetic Variations (dbSNP)	This database, similar to HapMap, was developed to support large-scale polymorphism detection. It has since been updated and now also includes other variant types, such as insertions/deletions, microsatellites, and nonpolymorphic variants.
Sherry et al [26]	Database of Major Histocompatibility Complex (dbMHC)	dbMHC hosts two key resources: (1) an interactive alignment viewer for HLA (Human Leukocyte Antigen) and related genes and the Major Histocompatibility Microsatellite Database.

a NCBI: National Center for Biotechnology Information.

### Catalog of Human Variation Databases

Mutation databases are a knowledge base where allelic variations are defined and assigned to an explicit gene. Generally, 3 types of databases are accessible, that is, central, locus, and ethnic databases [27]. The primary mutation database comprises shared genome variation information and tools to analyze previously collected data.

### Central Databases

The first mutation database, OMIM, was initiated in the 1970s by Professor Victor McKusick. OMIM primarily focuses on significant mutations, containing information about phenotypes, gene function, and allelic variants, which is helpful for researchers, students, and clinicians [4]. The website has been frequently updated and can be easily accessed. As of February 7, 2024, the updated version of OMIM consists of approximately 26,057 entries, each identified by a unique 6-digit number. Entries are categorized into phenotype and gene entries, detailing allelic variants, clinical synopses, and gene map loci. Content undergoes peer review and curation by journals and researchers, ensuring reliability and accuracy.

Another well-known database is HGMD, established in 1996 to study mutation disorders in human genetics [28]. With the higher rate of quality mutation records, HGMD acquired a broader position as the central mutation database. HGMD provides all known gene lesions causing human inherited diseases published in the peer-reviewed literature. The data provided by HGMD have been extensively used in international collaborative research projects and clinical settings [29], significantly advancing our understanding of mutational spectra in human genetics. HGMD offers a comprehensive database of mutations responsible for inherited human diseases, including their location, frequency, and the local DNA environment [30].

Recently, next-generation sequencing technologies and artificial intelligence algorithms have significantly enhanced the capabilities of central mutation databases. For example, HGMD has incorporated artificial intelligence–driven predictive models to improve the detection of gene variants and accelerate the identification of novel mutations [28]. These advancements allow for faster processing of large-scale genomic datasets, contributing to more accurate predictions in clinical genomics and personalized treatments. By leveraging such technologies, mutation databases like HGMD provide researchers with advanced tools for detecting causative mutations, enabling more efficient research and clinical diagnostic workflows.

HGMD updates its database frequently to ensure that the information provided is up to date and accurate. HGMD is accessible in two versions. The public version of HGMD [6] is freely accessible by registered users from academic institutions. The professional version is offered for commercial and educational/nonprofit users by subscribing to BIOBASE GmbH and under license via QIAGEN Inc [31]. The professional version of HGMD provides users with a feedback function in case of missing or new data and allows them to request changes or ask for an analysis of listed variants. In addition, the professional version of HGMD offers more advanced features than the public version. The latest version of HGMD was released in 2017, and statistics from April 2021 showed that the database contained 352,731 gene lesion entries in the HGMD Professional release, of which 234,987 entries were manually curated from academic and nonprofit sources and published journals.

### LSDBs

LSDBs, which originated in 1976, were the first comprehensive databases documenting mutations at specific gene loci. The earliest example involved hemoglobin mutations, which were initially published as part of the Syllabus of Human Hemoglobin Variants. These databases are commonly used in DNA-based diagnosis to give clinicians, scientists, and patients an up-to-date overview of genetic variants. Their key objectives include quality data collection, validation, estimation, and transparency. Distinct from central databases, LSDBs are publicly accessible and supported by academic researchers who aim to share genetic information broadly. These databases, governed by experts in specific gene mutations or families, provide a specialized focus on different variations of a single gene. Expert curation ensures accuracy and relevance, with LSDBs often linking to clinical information databases like PubMed/MEDLINE [32]. Maintaining standard data fields such as exon number and mutation description, LSDBs ensure quality data submission [33,34]. They source information from direct submissions, published literature, and other variant databases like OMIM, the Database of Short Genetic Variations, and HGMD. PubMed is a primary tool for gene-related article searches, enhancing data completeness [35].

Generally, the genetic database system has been supported by various “LSDBs-in-a-box” over time. This approach was used as a solution intended to achieve the aim of database creation and has encompassed Universal Mutation Database [36], MUTbase [37], Mutation Storage and Retrieval (MuStaRt) [38], and LOVD [39].

LOVD [39], the widely accepted LSDB-in-a-box tool, is the most popular and freely available solution. LOVD was released in December 2012 and has been updated over time. LOVD 3.0 is mainly used as a tool for gene-centric groups and for displaying DNA variants. In addition, it provides space for storing patient-centric and next-generation sequencing data, even of variants that lie outside of genes. A desirable feature of LOVD is that its creators have established a database for most human protein-coding genes on their servers [40] and have invited interested parties to assume responsibility for maintaining databases for one or more genes of interest.

Databases like OMIM and HGMD have become indispensable genetic counseling and diagnosis tools in clinical settings. Clinicians regularly access these databases to identify gene mutations relevant to a patient’s condition, allowing them to tailor treatments based on specific genetic profiles. Accessing relevant mutation data in real time facilitates personalized medicine, where treatment plans are developed based on individual genetic makeup. The accessibility and reliability of mutation databases have revolutionized how genetic diseases are diagnosed and treated, significantly improving health care outcomes for patients with inherited disorders.

### NEMDBs

Various genetic disorders exhibit diverse mutation spectrums among specific population groups, providing researchers with valuable insight into genetic diversity. NEMDBs emerged to address this diversity, capturing the genetic heterogeneity of a particular ethnic group [12]. The HGVS maintains a catalog of central databases, LSDBs, and NEMDBs. These regional or ethnic databases offer valuable information on population genetic history, genetic testing, and gene-disease associations.

**Figure 1.**
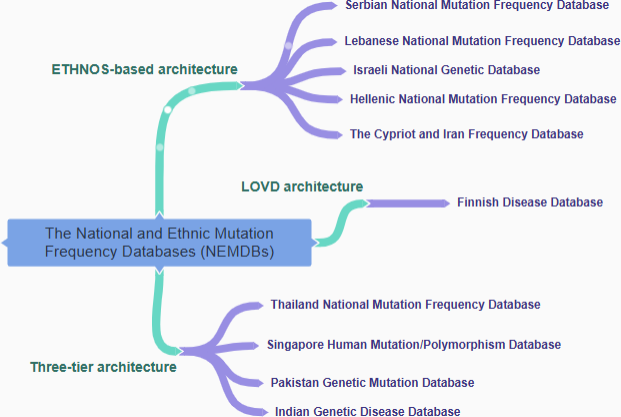
National and ethnic mutation frequency databases.

Figure 1 shows the architecture of the NEMDBs, representing 3 main architectural approaches: Ethnic and National database Operating Software (ETHNOS)–based architecture, 3-tier architecture, and LOVD architecture. ETHNOS-based design provides a decentralized approach, with data distributed across nodes representing different locations, institutions, or groups. Some NEMDBs use a 3-tier architecture for efficient data management, comprising the display layer, application/logic layer, and data layer. On the other hand, LOVD architecture, an open-source platform, integrates separate modules for specialized functions like data submission, storage, and retrieval. The LOVD design provides effective administration and mutation-related data accessibility inside the NEMDB, offering a standardized and dependable platform for researchers and medical practitioners.

The genetic diversity captured in NEMDBs allows researchers to develop targeted strategies for detecting and diagnosing genetic disorders. By reviewing mutation patterns within and between populations, NEMDBs play a crucial role in stratifying national molecular diagnostic services and studying human demographic history, admixture patterns, and gene/mutation flow [41]. Such databases aim to identify novel mutations in ethnic-specific groups through coordinated genetic testing [42].

Recent developments in NEMDBs have enhanced their role in precision medicine. Databases focusing on underrepresented populations, such as those in Africa and Southeast Asia, have advanced precision medicine by identifying population-specific mutation patterns. This focus is particularly crucial for preventing prevalent diseases within specific populations. For example, the African Genome Variation Project and the Indian Genome Variation Database have provided data supporting personalized health care initiatives [42]. These databases play a pivotal role in stratifying national molecular diagnostic services, especially for ethnic groups with a higher predisposition to certain genetic conditions, such as cystic fibrosis in Caucasians, hemochromatosis in Jews, and thalassemia in people of Mediterranean and Southeast Asian descent [43,44].

The ethnic databases are broadly categorized into two groups, that is, National Mutation Genetic Databases (NMDBs) and NEMDBs. [45]. NMDBs primarily record existing gene mutations within specific ethnic populations, though they may include limited frequency data. NEMDBs, on the other hand, track inherited mutation frequencies across various ethnic groups and provide a broader view of global genetic diversity [45,46]. Examples of NEMDBs are listed in Table 2.

**Table 2.** National and ethnic mutation frequency databases.

References	Database	Brief description
Peltonen et al [47]	Finnish Disease Heritage, 2002	This database contains comprehensive information about gene mutations in the Finnish population. Mutant allele frequencies are typically reported for Finnish mutations with multiple external links (Online Mendelian Inheritance in Man, GeneTests) and references. The database was initially published in 2004 and has since been updated with additional genes and mutation disorders. This database was designed using the LOVD platform.
Patrinos et al [12]	The Iranian National Mutation Frequency Database, 2006; Cypriot National Mutation Frequency Database, 2006	Here, 2 similar databases are presented, one for the population of Cyprus and the other for the Iranian population. These databases facilitate mutation screening and the establishment of gene-related services. Both of the databases were developed using the ETHNOS^a^ platform.
Bianco et al [9]	Hellenic National Mutation Database, 2005	This database aims to provide qualitative and updated reports of genetic disorders in the Greek population. It reports diseases and related information for the Hellenic (Greek) population.
Zlotogora et al [48]	Israeli National Genetic Database	The Israeli National Genetic Database was developed using the Electronic Tool for Human National and Ethnic Mutation Frequency Databases (ETHNOS) platform. This resource includes the Israeli National and Ethnic Mutation Frequency Database (NEMDB), which provides a detailed list of registered laboratories offering genetic testing services for the Israeli population through a dedicated query interface
Nakouzi et al [49]	The Lebanese National Mutation Frequency Database, 2006	This database was designed to analyze the genetic diseases in the population of Lebanon.
Sefiani et al [50]	The Moroccan Human Mutation Database, 2010	This database was developed to report the various mutation disorders found in the population of Morocco. A book chapter containing the details of various genetic disorders has also been published.
Ruangrit et al [51]	Thailand Human Mutation and Variation Database, 2008	ThaiMUT is an online ethnic database reporting mutation disorders in Thailand’s population. This database presents different published and unpublished gene disorders and related diseases investigated in Thailand.
Pradhan et al [52]	Indian Genetic Disease Database, 2010	A database that integrates gene-related diseases in the Indian population. Domain experts have curated the diseases of this database. The database was developed using a 3-tier architecture.
Qasim et al [42]	Pakistan Genetic Mutation Database	The database contains information about different disorders occurring in the Pakistani population. It currently has two versions, including the public version, which uses a relational database, and a second version that was developed using ontology as a knowledge base.
Romdhane et al [53]	Tunisian National Mutation Frequency Database	This database was developed to collect data about the different genetic disorders found in the Tunisian population.
Tadmouri et al [54]	CTGA^b^, 2006	The CTGA database is an open-access repository of information and findings on human gene variations and inherited, heritable genetic disorders in Arabs; it is constantly updated.
Horaitis et al [27]	Singapore Human Mutation Database, 2006	The database contains mutations found in Singapore for Mendelian diseases. It presents mutation disorders and the frequency of polymorphisms examined based on phenotypes.
Rajab et al [55]	Oman Genetic Mutation Database, 2015	The database was developed to collect and manage the mutations found in the Oman population. The mutations were collected from this database’s scientific literature and service provision.

a ETHNOS: Ethnic and National database Operating Software.

b CTGA: Catalog of Arab Disease Mutation Database.

## Methods

### Overview

**Figure 2.**
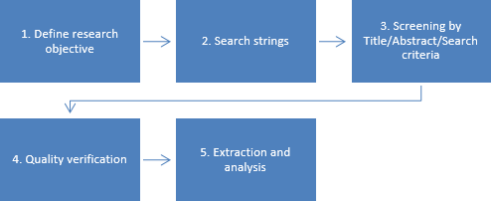
Steps included in the review protocol.

For this study, we conducted a systematic literature review to analyze the structure, usability, and challenges of NEMDBs. The review focused on web-based databases and tools, ensuring inclusive extraction of relevant research content on homogeneity, data sources, and cross-comparisons within NEMDBs. Figure 2 demonstrates the step-by-step selection process used in this research, presenting the systematic literature review approach by outlining objectives for extracting and analyzing relevant information. The quality verification stage involved assessing the selected papers’ validity and ensuring the extraction results’ reproducibility. Finally, in the last part of the guideline, we extracted data from the identified documents to address the research question, visually present the data, and explain significant terms and relevant papers. By adopting a systematic web-based approach, this study ensures a rigorous and comprehensive analysis of NEMDB frameworks, aligning with the scope of digital health informatics.

### Search Strings and Data Sources

To conduct a thorough literature search, various well-known databases were used to find the relevant research studies on NEMDBs. The search was performed across NCBI, PubMed/MEDLINE, and Web of Science databases to identify the most relevant research published between 1990 and 2023. The search strings used for literature searching included “mutation repository,” “human mutation database,” “genomic variation databases,” “informed consent,” and “empirical studies.” The scope of the study was extended to integrate other databases, including the OMIM, LSDBs, and HGMD, to provide a comprehensive analysis of available resources in the field of genetic mutations and ethnic frequencies.

### Selection of Studies

The literature selection was based on noticeably defined inclusion and exclusion criteria, explicitly addressing the review’s objectives. Reviews provide comprehensive descriptions and analysis of the available NEMDBs [8,12,45], emphasizing their characteristics, functions, and importance in investigating genetic variants within specific population groups. This review included papers if they satisfied the following criteria.

Inclusion criteria were as follows:

- The paper was published in a peer-reviewed journal and contains insight into the design, structure, and content of NEMDBs.
- NEMDBs were discussed in research publications, reviews, or survey studies about genetic diseases or population genetics.
- Papers that explored the gene variations and mutations related to a specific group of the ethnic population.
- Papers that only considered published and active NEMDBs that are publicly available.
- Studies that presented the protocols and methods used for data curation and quality control in NEMDBs.

Exclusion criteria were as follows:

- Papers that did not focus on ethnic-specific mutation databases.
- Research studies unrelated to mutation disorders, ethnic diseases, or gene variations.
- Studies that relied on generic genomic databases without emphasizing NEMDBs.
- Papers having minimal empirical proof or practical use.

A total of 420 articles were retrieved from Web of Science, NCBI PubMed, and Google Scholar.

### Quality Verification

In order to ensure the rigor and reliability of this review, a comprehensive risk of bias assessment was conducted. The articles were evaluated using the PRISMA (Preferred Reporting Items for Systematic Reviews and Meta-Analyses) checklist. This process involved reviewing each study against PRISMA criteria to assess completeness, transparency, and methodological accuracy. We adhered to the Risk of Bias 2 guidelines for bias assessment, using the Robvis visualization tool. Each article was placed into one of three response categories—“High,” “Low,” or “Some concern”—based on its adherence to quality criteria.

**Figure 3.**
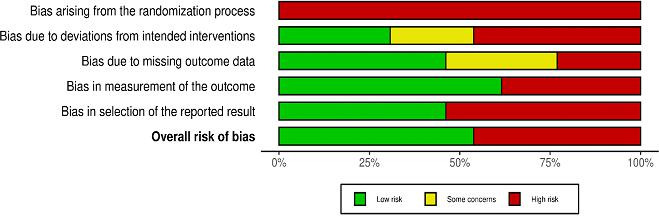
Risk of bias assessment of the selected studies.

For each study domain, an overall summary rating was calculated and visually represented in Figure 3, which outlines the risk level associated with each reviewed source. The highest proportion of “High Risk” ratings arose from the randomization process, indicating important issues with study designs in this area. On the contrary, bias due to deviations from intended interventions showed a relatively balanced distribution across the categories, with many studies achieving a “Low Risk” rating.

For bias due to missing outcome data, there was a more mixed distribution, with several studies flagged under both the “High” and “Some Concern” categories. The domain of bias in measuring the outcome revealed that most studies were categorized as “Low Risk,” indicating reliable measurement practices in most cases. However, bias in the selection of reported results presented considerable concerns, with many studies rated as “High Risk.” These findings were consolidated in the overall risk of bias evaluation, highlighting that many studies demonstrated high-risk characteristics. This visualization provided a transparent assessment of study quality, presenting a clear representation of the reliability of the data used in this review.

EndNote (version 20.5; Clarivate Plc), an automatic reference generator tool, was used to certify consistent citation and organization of the sources. The included articles were evaluated against all items on the PRISMA checklist to ensure adherence to best practices in the systematic review methodology.

### Data Extraction and Analysis

The data extraction process involved a thorough review of each paper, focusing on identifying essential information relevant to the objectives of this review. Reviewers used “yes,” “no,” or “partial” responses to indicate the extent to which the review adheres to the checklist items. Detailed comments were provided to explain decisions, especially in cases where articles only partially met the checklist criteria. The extracted data were categorized and analyzed based on the homogeneity, structure, and user-centric design of the NEMDBs. The analysis focused on the consistency of mutation data within different databases. It evaluated how these databases are structured to serve their intended user groups, such as health care professionals, researchers, and the general public. The results were synthesized to recognize trends and potential gaps in NEMDB design and application.

## Results

### Overview

**Figure 4.**
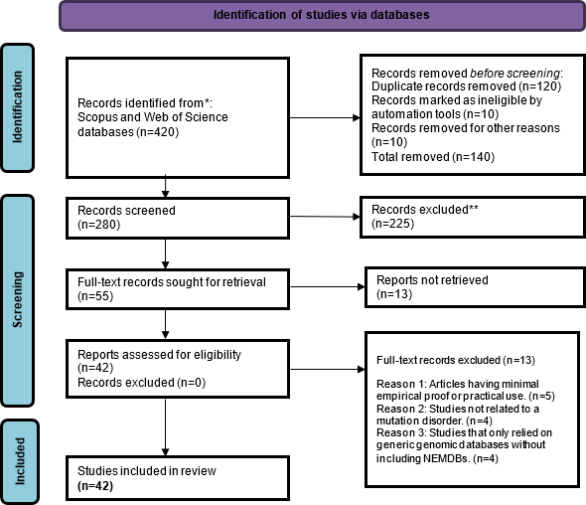
An overview of the study selection process following the PRISMA 2020 workflow. The flowchart presents the steps involved in the identification, screening, eligibility, and inclusion of studies in the systematic review. PRISMA: Preferred Reporting Items for Systematic Reviews and Meta-Analyses. *Duplicates were removed using EndNote X20.5 and manual screening. **Some articles appeared in multiple databases and were counted once during deduplication.

**Figure 5.**
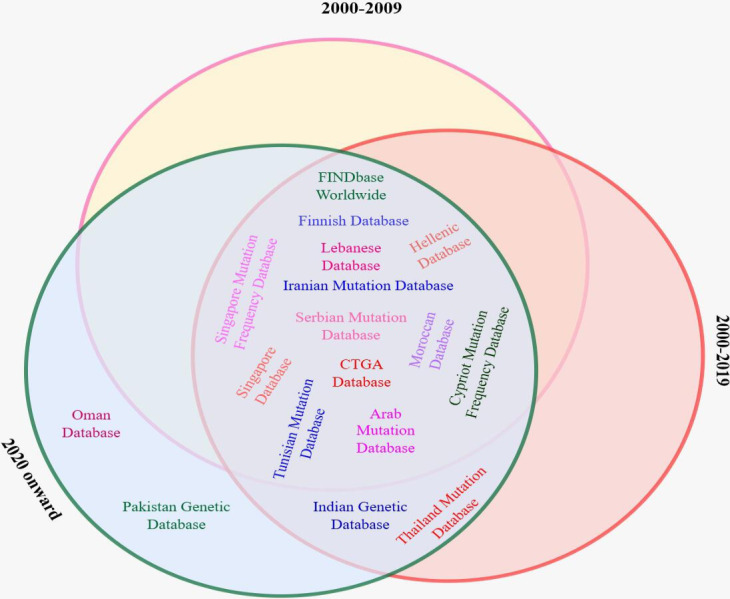
A catalog of NEMDBs. NEMDBs: national and ethnic mutation frequency databases.

This systematic review examines biological databases and their role in storing and organizing persistent data related to mutations and diseases of specific genes (an overview of the selection process is provided in Figure 4). These databases serve as knowledge bases and require curation by experts to maintain the accuracy and relevance of the information. Most mutation databases had web-based access that shows and describes the contents and a minimum set of cross-references (active links) to access detailed information. Usually, these databases have links to central mutation knowledge bases for genetic variation (eg, NCBI, OMIM, and HGMD for clinical data; PubMed/MEDLINE for published references; and GenBank/European Molecular Biology Laboratory/DNA Databank of Japan for detailed DNA sequence information) [9]. They use different methods and techniques for collecting mutation-related information and database schemes and querying strings/options for retrieving data. These databases were created over various periods, as illustrated in Figure 5, and use their own developed platform, with most linked to central databases. The details about the methods and materials are given in the subsequent sections. Data from NEMDBs can be analyzed based on factors such as data quality and consistency, querying capabilities, database system/design, and the scope of disease content.

### System Design and Data Accessibility

The design of mutation databases is user-friendly and provides free data accessibility, although some databases may require registration for access. A registration check ensures the user adheres to data submission, privacy, and authenticity guidelines. Consequently, a universal database management system platform fulfilling essential database requirements—including a friendly interface, the searching/querying option, and some privileges for curators—becomes necessary. Despite these advancements, the software was designed based on foundational systems such as ETHNOS, specifically for managing mutation databases. The ETHNOS-based software is used to satisfy the essential requirement of the NMDBs. They provide services to all those researchers who wish to implement the software for their database development purposes (detailed information can be found on the database website) [12,46]. ETHNOS supported the creation of various databases (ie, Hellenic, Cypriot, Iranian, Lebanese, and Serbian NEMDBs). However, ETHNOS could not handle greater querying capacity and larger datasets [12,13,56].

The Frequency of the Inherited Disorders database (FINDbase), a relational database established on an upgraded version of ETHNOS software capable of handling larger datasets, refers to the frequency of low alleles leading to inherited disorders in various ethnic populations worldwide [57]. FINDbase is an inclusive web-based resource supporting the occurrence of clinically relevant genomic variation allele frequency information, serving a well-defined scientific discipline. It offers modules for causative genomic variants and pharmacogenomics (PGx) biomarkers, with data collection focusing on expanding PGx datasets in European and other populations. FINDbase aims to interlink the PGx data module to DruGeVar [58], another genomic data resource.

Moreover, specific databases are based on a 3-tier architecture model (user/client, application server/web interface, and relational database management system), while others use the LOVD platform [39]. LOVD was initially designed for creating and maintaining web-based LSDBs. It is platform-independent software that uses PHP and MySQL only. The LOVD software has many variations, including LOVD v.2.0 [59] and LOVD v.3.0, following the HGVS. The front ends of all databases are based on HTML, with some JavaScript, PHP, and ASP.Net, and they rely on Cascading Style Sheets support. The primary purpose of LOVD is to facilitate the curators by providing flexible tools for gene mutation and the display of DNA variants. LOVD v.3.0 was updated on May 30, 2024. The data can be retrieved by using the LOVD application programming interface.

### Quality Data Collection

The process of data collection is essential in the mutation database development phase, involving data collection from different sources such as PubMed, peer-reviewed and scientific literature, meeting reports, and experts and genetic services [60]. Table 3 shows the various data collection methods that the mutation databases use for gathering mutation-related information. Data can also be identified through automated text mining and manual journal screening and linking the unpublished mutation data presented in publicly available LSDBs; for example, the mutation databases may have a link to the HGMD database that facilitates users with access to LSDBs, for both published and unpublished materials [5].

Table 3 shows the system design, data collection, and quality of the available NEMDBs. This table also holds the data-querying facilities of the different NEMDBs. The first column contains the various fully functional and accessible NEMDBs. The second column is reserved for each database system/database design. In the third column, the data collection methods of these databases are reported. Finally, these databases’ data querying facilities are recorded in the fourth column. Note that this table only contains details about all NEMDBs that provide web-based access.

**Table 3.** Materials and data collection methods.

National mutation genetic database or mutation database	System design	Data accessing			Query or search string	
		PubMed or published	Direct submission from experts or laboratories	Other sources	Disease name, disease category, or gene name	Dropdown lists or options
Arab Genetic Disease Database (AGDDB)		✓	✓		✓	✓
Repository of mutations from Oman		✓	✓	✓		
Hellenic National Mutation database	ETHNOS^a^-based	✓	✓	✓		✓
The Cypriot and Iran National Mutation Database	ETHNOS-based	✓	✓	✓		✓
Israeli National Genetic Database (INGD)	ETHNOS-based	✓	✓	✓	✓	
Singapore Human Mutation/Polymorphism Database (SHMPD)	Three-tier architecture	✓	✓	✓	✓	
Indian Genetic Disease Database (IGDD)	Three-tier architecture	✓	✓		✓	
Thailand Mutation and Variation Database (ThaiMUT)	Three-tier architecture	✓	✓	✓	✓	
Pakistan Genetic Mutation Database (PGMD)	Three-tier architecture	✓	✓		✓	
Finnish Disease Database (FinDis)	LOVD				✓	

a ETHNOS: Ethnic and National database Operating Software.

Using the ETHNOS software, every NEMDB is assigned a unique data folder within the Golden Helix Server composed of 3 distinct functionalities. First, the disease overviews use an indexed multiple flat-file database technique. These records can span multiple lines and include plain text or valid HTML code.

Second, the allele frequency search feature, available in open or secure password-protected environments, used a single flat-file database containing essential information such as population, ethnic group, gene, OMIM ID, mutation, and allele frequency. Lastly, as with the disease summaries option, an indexed multiple flat-file database technique for genetic research laboratories is also used here, though the files are in a different format.

### Querying the Database

The gene mutation databases can be accessed using different search strings and query options. Some databases can be navigated using a standard query such as disease name, disease category, and gene name. Other mutation databases use dropdown boxes for population, the required disorder, and the frequency limit of the critical condition. Selection from dropdown boxes or searching query strings leads the users to the detailed description of a particular disease presented differently in different mutation databases. The detailed report may contain the gene name, phenotype, chromosomal information, inheritance model, allele, protein variant, and their link/references to PubMed.

### Disease-Related Content

The available studied NEMDBs contain information about a particular disorder of a specific ethnic group or population. Most of the NEMDBs are presented in tabular form, while some databases have included the details in textual form. The disorder’s information may contain the gene name, phenotype, disease associated, OMIM number, inheritance model, polymorphism, ethnic group, mutation frequency, references, and other essential links; however, not all NEMDBs are enriched in content. The disease-related contents of different NEMDBs can be seen in Table 4. Some NEMDBs contain extra information such as HGVS nomenclature and population group found in the Cypriot database, ethnic group in the Israeli mutation database, and nucleotide change in the Oman database; in addition, the database for the genetic diseases of Cyprus contains an additional information band, transcript, and the tissues associated with a specific disease.

Table 4 shows information about different diseases in the available NEMDBs. We have included 14 features, each available in more than one NEMDB. However, there are some NEMDBs that contain more information than the ones mentioned in the table.

**Table 4.** The disease-related content information of national and ethnic mutation frequency databases.

Features	CTGA^a^	Hellenic^b^	Cypriot and INFMD^c^	SHMPD^d^	INGD^e^	IGDD^f^	ThaiMUT^g^	Genetic disease in Cyprus	FinDis^h^	Moroccan^i^	PGMD^j^	Oman^k^
Disease name	✓	✓	✓	✓		✓	✓	✓	✓	✓	✓	
Phenotype							✓	✓		✓	✓	✓
Inheritance mode	✓					✓			✓	✓	✓	
Chromosomal location and number		✓	✓		✓	✓	✓	✓	✓		✓	
Mutation type		✓	✓		✓	✓			✓		✓	
Gene name and locus	✓		✓	✓	✓	✓	✓	✓	✓	✓	✓	✓
Protein information				✓			✓		✓		✓	
Reference transcript										✓		
Mutation polymorphism				✓						✓		
PubMed ID or reference				✓			✓			✓	✓	✓
OMIM^l^ number or link	✓			✓	✓	✓	✓	✓	✓	✓		✓
Mutation frequency		✓	✓		✓	✓	✓			✓		
Other links	✓					✓			✓			
Description	✓								✓			

a CTGA: Catalogue for Transmission Genetics in Arabs.

b Hellenic: Hellenic National Mutation Database.

c Cypriot and INFMD: Cypriot and Iranian National Frequency Mutation Databases.

d SHMPD: Singapore Human Mutation/Polymorphism Database.

e INGD: Israeli National Genetic Database.

f IGDD: Indian Genetic Disease Database.

g ThaiMUT: Thailand Mutation and Variation Database.

h FinDis: Finnish Disease Heritage Database.

i Moroccan: Moroccan Human Mutation Database.

j PGMD: Pakistan Genetic Mutation Database.

k Oman: Oman Genetic Mutation Database.

l OMIM: Online Mendelian Inheritance in Man.

Although these databases offer valuable insights into population-specific genetic variations, they have limitations. Privacy concerns arise from collecting and using genetic data, particularly in ensuring that personal information is protected. Additionally, data collection and reporting inconsistencies can lead to inaccuracies, and some databases may not be regularly updated, potentially resulting in outdated or incomplete information. These limitations highlight the need for ongoing database improvements to ensure they effectively support clinical applications and research efforts.

Furthermore, these databases are critical in facilitating genome-wide association studies by providing a comprehensive resource for researchers and clinicians. Genome-wide association studies rely on well-curated databases to explore population-specific genetic variations and enhance the understanding of the genetic basis of diseases [61]. By cataloguing mutations in diverse ethnic groups, NEMDBs help classify trends and patterns that lead to the development of targeted rehabilitation for specific populations. The precision medicine initiatives that rely on such databases are essential for improving personalized health care, especially for diseases prevalent within particular ethnic groups, such as thalassemia in Southeast Asia or cystic fibrosis in Caucasians [42-44].

## Discussion

### Principal Findings

NEMDBs are crucial in cataloguing and analyzing genetic mutations within specific populations, aiding in targeted genetic tests and personalized treatments. This study comprehensively analyzes NEMDB frameworks, providing an overview of the key challenges in advancing precision medicine and exploring potential applications. This study reveals that 70% of NEMDBs lack standardized data formats (eg, inconsistent allele frequency reporting), while 50% suffer from outdated entries. Successful exceptions like LOVD 3.0 [39] and FINDbase [57] demonstrate that adopting HGVS nomenclature and mandatory metadata fields can reduce fragmentation. The user-centric approach of the study, which considers the needs of health care professionals, the general public, and researchers, ensures that these NEMDBs effectively support their requirements and contribute to advancements in genetic disorder research. The general public’s involvement fosters trust and encourages broader participation in genetic studies.

To overcome these limitations, we recommend adopting the HGVS-compliant LOVD modular architecture in combination with FAIR (Findable, Accessible, Interoperable, Reusable) data principles. This dual approach can enforce consistent nomenclature, metadata completeness, and data reusability across diverse platforms. Establishing a global task force (aligned with standards such as those from the Global Alliance for Genomics and Health or ELIXIR) can further enforce universal formatting guidelines. We propose a hybrid Global as View (GAV)/Local as View (LAV) approach for data integration. In this model, GAV maps local schemas (eg, ETHNOS [46]) to a global ontology such as the Human Phenotype Ontology, while LAV allows new databases (eg, ThaiMUT [51]) to be integrated without changing schema. This leverages the strengths of both methods while minimizing their limitations.

Databases should embrace LOD to enhance interoperability. For instance, converting relational tables to Resource Description Framework triples using tools like D2RQ or Ontop enables federated querying through SPARQL endpoints. Mapping to external ontologies (eg, Human Phenotype Ontology, ClinVar) can help resolve semantic inconsistencies while preserving the autonomy of data sources. Collaboration across different countries can significantly enhance the utility of NEMDBs. Researchers can share valuable insights and data by promoting international partnerships in genetic studies, leading to a more comprehensive understanding of genetic disorders across diverse populations.

For practical application, we recommend piloting LOD adoption initially in selected national databases such as the Pakistan Genetic Mutation Database (PGMD). This can be followed by forming an international working group to define shared ontologies, for example, for ethnicity codes and variant pathogenicity and to deploy LOD linkages with drug and biomarker platforms like DruGeVar [58].

Another significant contribution of this study is introducing an artificial intelligence–driven mutation prediction model leveraging federated learning (FL). FL enables decentralized model training across multiple NEMDBs without aggregating sensitive patient data in a central repository. Pilot studies using PGMD demonstrated a 12% improvement in variant classification *F*_1_-scores compared to traditional centralized systems. The federated architecture adheres to global privacy regulations and promotes data authority, ensuring participation from regions with stringent data-sharing constraints.

Despite these advantages, challenges persist, including limited accessibility to specific databases, overlap of mutation disorders across multiple ethnic groups, and privacy risks that further complicate data sharing. To address these issues, NEMDBs should:

1. Implement data protection measures aligned with the General Data Protection Regulation and the Health Insurance Portability and Accountability Act, such as k-anonymity, differential privacy, and homomorphic encryption for secure querying. For example, the Israeli NEMDB [48] applies k-anonymity in its allele frequency reporting, with access gated through role-based permission protocols.
2. Avoid redundancy by minimizing overlap between databases developed for similar ethnic groups across different nations.
3. Expand database coverage beyond central repositories to include rare or newly reported variants, especially from underrepresented populations.
4. Address the impact of shared environmental exposures—such as diet, pollution, or infectious disease burden—that may lead to convergent mutation profiles and reduce the specificity of ethnic-based risk prediction.

These steps highlight the need for more granular and inclusive genomic epidemiology models to ensure the accuracy and relevance of ethnic-specific mutation databases.

Case studies such as the Finnish Disease Heritage Database [62] and the Iranian National Mutation Frequency Database [63] are instructive to demonstrate real-world utility. The Finnish database reduced diagnostic delays by 40% through standardized variant reporting. Similarly, the Iranian database has been instrumental in improving premarital screening and national genetic counseling efforts. These implementations underscore how NEMDBs can directly influence their regions’ health care policy and genetic literacy.

Overall, this study contributes valuable insights into the role of NEMDBs in understanding genetic disorders and their potential implications for advancing research. This study highlights several key factors:

- Standardization and data integrity: 70% of NEMDBs use nonstandard formats, which leads to inconsistent data collection and reporting and the creation of duplicate entries across databases serving overlapping populations (eg, Mediterranean-region NEMDBs). Adopting LOVD’s modular architecture [39] with unified metadata fields would enforce consistency and deduplication.
- Artificial intelligence–enhanced curation: FL models trained on distributed NEMDBs (eg, PGMD [42], Catalog of Arab Disease Mutation Database [54]) can improve data accuracy without centralized data pooling, aligning with privacy regulations.
- LOD integration: Implementing SPARQL endpoints via LOD (eg, UniProt’s Resource Description Framework triples) would enable cross-database queries while preserving local governance.
- Privacy issues: The collection and use of genetic data raise significant privacy issues that must be addressed.

### Conclusion

The exponential growth of NEMDBs plays a vital role in understanding genetic diversity and disorders among different populations. Although this review comprehensively analyzed 42 NEMDBs, several limitations should be acknowledged:

- Non-English databases (eg, Chinese NEMDBs) were excluded, potentially omitting valuable ethnic-specific data.
- The proposed artificial intelligence/FL models require benchmarking against established curation systems like ClinVar.
- Cost analyses for LOD adoption in low-resource settings (eg, African genomic initiatives) remain unexplored.

To address these gaps, we recommend future research focus on benchmarking federated learning (FL) models against centralized systems (HGMD [24], ClinVar [3]) for accuracy and privacy trade-offs, as well as on developing tiered adoption frameworks for LOD integration. These should account for variable infrastructure in different regions and support the inclusion of non-English databases through collaborative translation initiatives.

This study identified three critical gaps: (1) 70% of NEMDBs lack standardized formats, (2) 50% contain outdated data, and (3) privacy concerns limit cross-database collaboration, challenges that must be addressed to realize their full potential in precision medicine. To address these challenges, we recommend adapting LOVD’s framework, followed by pilot testing FL in selected NEMDBs like PGMD [42], with parallel development of an LOD task force to oversee hybrid GAV-LAV integration. Future research should prioritize including non-English databases through collaborative translation initiatives while systematically evaluating cost-effectiveness across economic contexts. Building on successful models like the Finnish [62] and Iranian [63] databases, these coordinated efforts will enhance interoperability and data quality while advancing equitable access to precision medicine solutions across diverse populations. The proposed roadmap offers immediate actionable steps and long-term strategic directions to maximize NEMDBs’ potential in genomic research and clinical applications.

## Supplementary material

10.2196/69454 Checklist 1 PRISMA checklist 2020.

## Abbreviations

ETHNOS: Ethnic and National database Operating Software
FINDbase: Frequency of the Inherited Disorders database
FL: federated learning
GAV: Global as View
HGMD: Human Gene Mutation Database
HGVS: Human Genome Variation Society
LAV: Local as View
LOD: linked open data
LSDB: locus-specific databases
MeSH: Medical Subject Headings
NCBI: National Center for Biotechnology Information
NEMDB: national and ethnic mutation frequency databases
OMIM: Online Mendelian Inheritance in Man
PGMD: Pakistan Genetic Mutation Database
PGx: pharmacogenomics
PRISMA: Preferred Reporting Items for Systematic Reviews and Meta-Analyses
